# Differential Effects of Short Term Feeding of a Soy Protein Isolate Diet and Estrogen Treatment on Bone in the Pre-Pubertal Rat

**DOI:** 10.1371/journal.pone.0035736

**Published:** 2012-04-20

**Authors:** Jian Zhang, Oxana P. Lazarenko, Xianli Wu, Yudong Tong, Michael L. Blackburn, Horatio Gomez-Acevedo, Kartik Shankar, Thomas M. Badger, Martin J. J. Ronis, Jin-Ran Chen

**Affiliations:** 1 Arkansas Children's Nutrition Center, Little Rock, Arkansas, United States of America; 2 Department of Pediatrics, University of Arkansas for Medical Sciences, Little Rock, Arkansas, United States of America; 3 Department of Physiology and Biophysics, University of Arkansas for Medical Sciences, Little Rock, Arkansas, United States of America; 4 Department of Pharmacology and Toxicology, University of Arkansas for Medical Sciences, Little Rock, Arkansas, United States of America; IIT Research Institute, United States of America

## Abstract

**Background:**

Previous reports suggest that beneficial effects of soy on bone quality are due to the estrogenic actions of isoflavone phytochemicals associated with the protein. However, mechanistic studies comparing the effects of soy diet and estrogens on bone, particularly in rapidly growing animals are lacking.

**Methodology and Principal Findings:**

We studied the effects of short term feeding of soy protein isolate (SPI) on bone in comparison to the effects of 17β-estradiol (E2) in pre-pubertal rats. Female rats were weaned to one of 4 treatments: 1) a control casein-based diet (CAS); 2) CAS with subcutaneous E2 (10 µg/kg/d) (CAS+E2); 3) a SPI-containing diet (SPI); or 4) SPI with subcutaneous E2 (SPI) or SPI with 10 µg/kg/d E2 (SPI+E2) for 14 days beginning on postnatal day 20. SPI increased while E2 decreased bone turnover compared to CAS. In contrast, both treatments decreased serum sclerostin levels. Microarray analysis of RNA isolated from bone revealed 652 genes regulated by SPI, 491 genes regulated by E2, and 266 genes regulated by both SPI diet and E2 compared to CAS. The expression of caveolin-1, a protein localized in the cell membrane, was down-regulated (p<0.05) in rats fed SPI, but not by E2 compared to rats fed casein. Down-regulation of caveolin-1 by SPI was associated with increased BMP2, Smad and Runx2 expression in bone and osteoblasts (p<0.05).

**Conclusions/Significance:**

These results suggest SPI and E2 have different effects on bone turnover prior to puberty. Approximately half of the genes are regulated in the same direction by E2 or SPI, but in combination, SPI blocks the estrogen effects and returns the profile towards control levels. In addition, there are E2 specific and SPI-specific gene changes related to regulation of bone formation.

## Introduction

Although beneficial skeletal effects of soy-containing diets have been suggested for decades [1], this is a controversial area. In addition, the mechanisms of potential soy effects on bone remain unclear, but most reports suggest that soy phytochemicals (isoflavone phytoestrogens, such as genistein and daidzein) are responsible. Isoflavones are structurally similar to 17β-estradiol and are estrogenic under certain physiological conditions [2]. Therefore, soy has been used by some postmenopausal women as a natural alternative to estrogen replacement therapy [3]. More recently, evidence suggests that isoflavones may not only have estrogenic effects, but are also antiestrogenic in some mammalian tissues [4]. The preferential binding of isoflavones to estrogen receptor β suggests that they may act as SERMs (selective estrogen receptor modulators) [5]. The majority of soy diet and bone research has been conducted in adult human participants and experimental rodents. However, unlike in some Asian countries where soy diets are constantly consumed by every age group in the population, in the USA, soy-containing food is mainly consumed by infants in the form of soy infant formula [6]. Assessing the effects of soy consumption early in life on estrogen sensitive organs has become a high research priority as the result of safety concerns with regards to potential toxic effects on reproductive development and increased cancer risk [7], [8]. We and others have started to examine the effects of soy foods consumed in early life and evaluated its consequences on bone health later in adult life [9], [10]. We hypothesized that dietary factors other than isoflavones appearing in serum after feeding soy diets may also have effects on bone formation. The relative concentrations and forms of dietary factors associated with soy diets are significantly affected by processing of soy beans into different products, and may be species and age dependent [11].

Human peak bone mass is usually reached in the late third decade of life. This bone mass or mineral density in adults reflects the extent of bone acquisition during growth and affects the subsequent rate of bone loss [12]. More than half of adult bone mass is made under the influence of growth hormone from the anterior pituitary gland and sex hormones during the peri-pubertal period [13]. Appropriate bone modeling during the peri-pubertal period may be essential to determine whether an individual is at higher or lower risk for bone fracture later in adult life [14]. Although the exact mechanisms and differences between males and females are not well understood, estrogen interacting with growth hormones is absolutely critical in both sexes for building bone strength and increasing long bone length. In rodents, such as rats, bone growth from weaning to postnatal day 35 is rapid and is under the control of a variety of hormones and cytokines. Bone mass acquisition during this time is similar to conditions which occur in pre-pubertal humans. Whether soy phytochemicals interact with the endogenous factors to determine: bone structure; bone turnover; and homeostasis of calcium, phosphorus and vitamin D during the pre-pubertal period are not well understood.

It is well known that the actions of estrogen in a variety of tissues and cells are primarily through estrogen receptors alpha (ERα) and beta (ERβ). While these receptors have overlapping responsibilities, they also have distinct roles in different tissue and cell types [15]. It has been established that one of the effects of estrogen on bone cells is through classic activation of ERα in the nucleus to transcribe estrogen-responsive genes. In contrast to this classic estrogen signaling in bone cells, previous studies suggest that a membrane ER complex may also activate cytoplasmic kinases to transmit non-genomic actions of estrogens [16]. Interestingly, soy consumption has been shown to activate cytoplasmic kinases such as ERK and Smad(s) in bone in vivo, which are requirements for osteoblast differentiation/osteoblastogenesis [9]. We have previously hypothesized that the effects of soy diet on bone may be largely due to non-estrogenic actions [17]. The purpose of the present study was to compare the short term effects of consumption of a soy diet relative to 17β-estradiol on bone turnover in prepubertal intact female rats. In addition, studies were conducted to investigate signaling transduction to determine whether the effects of SPI (soy protein isolate) feeding on bone cells are different from classical actions of estrogens.

## Results

### Effects of SPI and E2 treatment on serum isoflavone and E2 concentrations

Measurement of serum isoflavone values after feeding SPI confirmed previous data from rats in our laboratory (Table 1) [18]. Total isoflavone concentrations were in the range of 1 µM and the major isoflavone was the most estrogenic daidzein metabolite equol (Table 1). In addition, more than ninety percent of isoflavones were found in the conjugated form and free aglycone values were less than 40 nM. E2 treatments increased mean serum E2 concentrations from 25±3 pM in controls to 71±6 pM in the 10 µg/kg/d group. Addition of SPI had no significant effect (by t-test) on serum E2 values either when fed alone (36±6 pM SPI vs 25±3 pM CAS) or when fed in combination with 10 µg/kg/d E2 (71±6 pM, CAS+10 µg/kg/d E2 vs 91±14, SPI+10 µg/kg/d E2).

**Table 1:** Serum isoflavone concentration.

Serum isoflavone levels (nM)				
	Control		SPI	
	Free	Total	Free	Total
DHD	nd	nd	1.2±0.19	140.1±26.2
Glycetein	nd	nd	9.5±0.7	60.0±4.9
Genistein	nd	nd	38.2±9.6	921.8±80.7
Daidzein	nd	nd	37.4±8.7	521.6±50.8
o-DMA	nd	nd	5.4±0.31	767.4±88.8
Equol	nd	nd	15.7±2.5	2207.4±193.8

Serum isoflavones were measured using LC-MS method.

nd, non detected. DHD, dihydrodaidzein. o-DMA, o-desmethylangolansin.

### Effects of SPI and E2 on Bone Turnover in Prepubertal Rats

**Figure 1:**
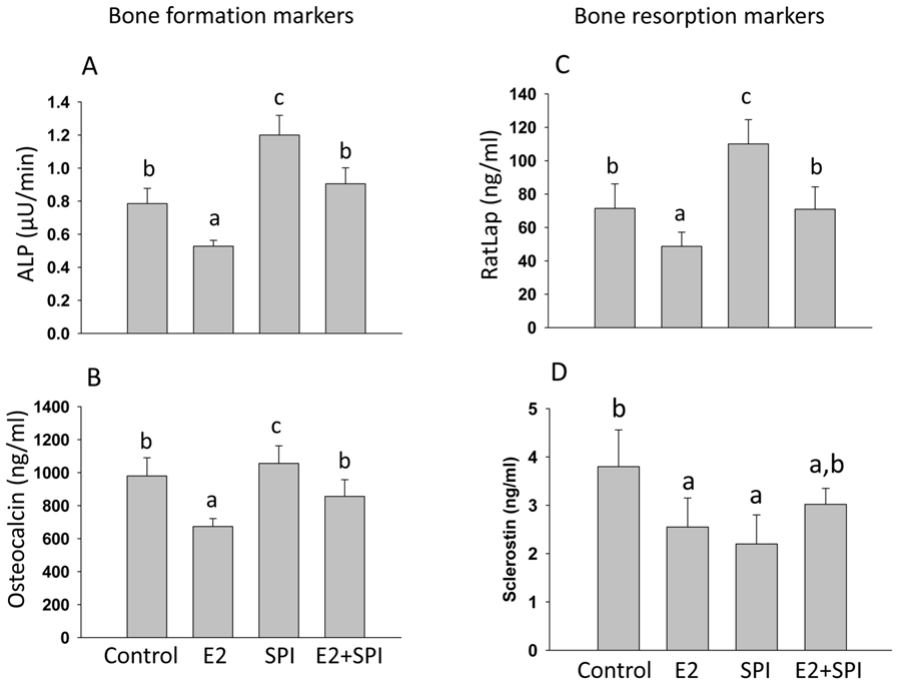
Changes in bone resorption and formation markers in pre-pubertal rats. (A) Serum bone formation markers ALP (bone specific alkaline phosphatase) and (B) osteocalcin, and (D) serum level of sclerostin were measured by ELISA. (C) Bone resorption marker RatLap (C telopeptide of type 1 collagen) was measured using a CrossLaps ELISA kit. Control, standard casein diet group; E2, 10 µg/kg/d E2 treated group; SPI, soy protein isolate diet group; E2+SPI, combination of 10 µg/kg/d E2 treated and SPI diet group. Means ± S.E.M with different letters differ significantly from each other at p<0.05, a<b<c.

Bone turnover markers were measured in rat serum using ELISA. The bone resorption marker RatLap and bone formation markers ALP and osteocalcin were lower (p<0.05) in the CAS+E2 group compared to CAS, the bone resorption marker RatLap and bone formation markers ALP and osteocalcin were all higher in SPI compared to CAS groups (p<0.05, Figure 1A–C). Interestingly, circulating levels of sclerostin were lower in both SPI and CAS+E2 groups compared to CAS (p<0.05, Figure 1D).

### Changes in gene expression in bone in response to SPI and E2

**Figure 2:**
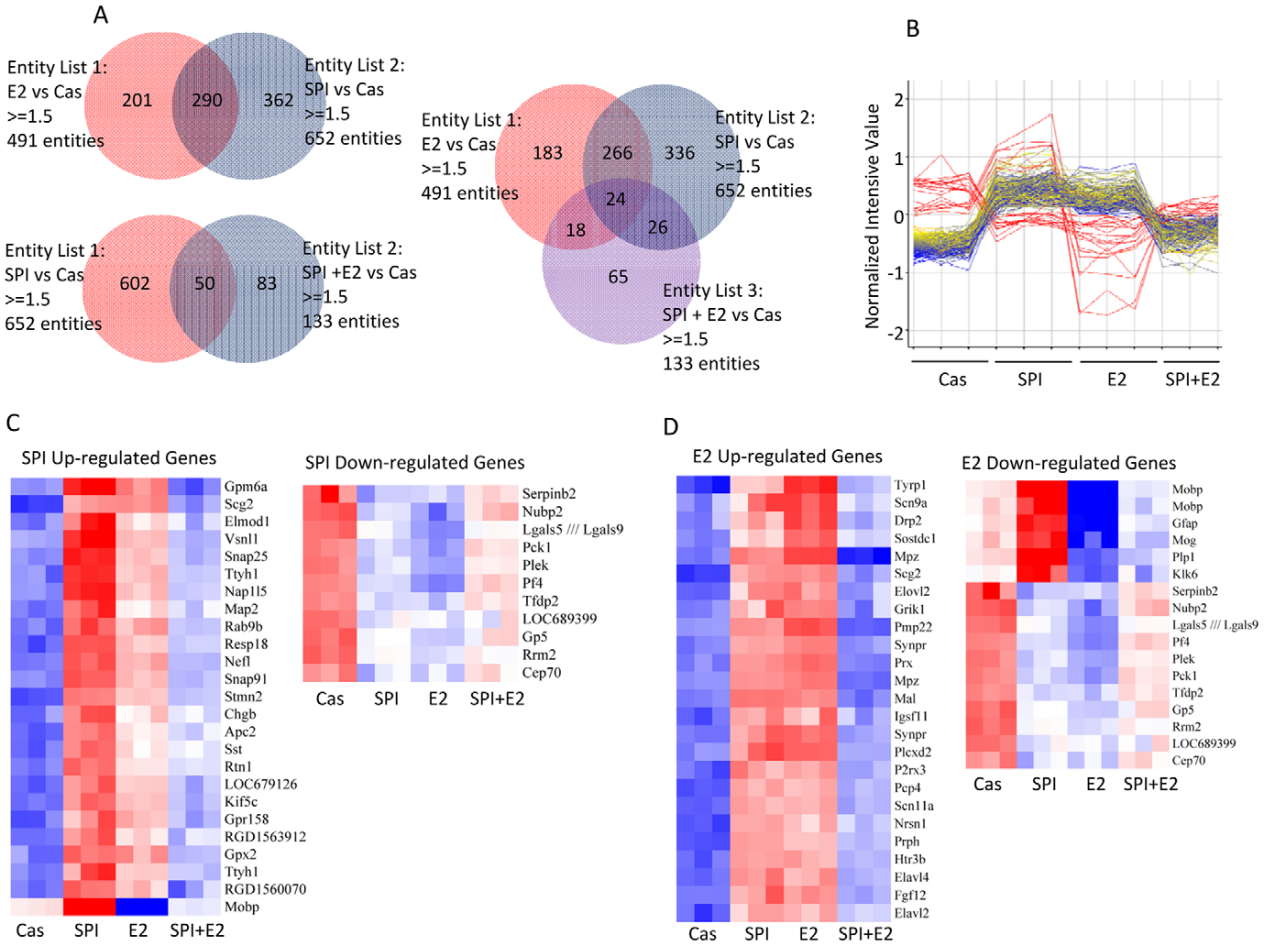
Microarray analysis shows differential signatures of SPI, E2, and SPI+E2 on gene expression in bone in pre-pubertal female rats. (A) Venn diagrams on the differentially expressed genes among groups. (B) Different pattern of gene regulation by SPI, E2 and SPI+E2 from 266 genes showed in (A). (C) Top 25 genes up-regulated by SPI and all genes down-regulated by SPI from 266 genes. (D) Top 25 genes up-regulated by E2 and all genes down-regulated by E2 from 266 genes showed in (B).

To identify the target genes in bone interacting with SPI or E2 that result in their different effects on bone, we analyzed mRNA from long bone using Affymetrix gene arrays. These analyses revealed 652 genes regulated more than 1.5-fold by SPI diet, 491 genes regulated by 10 µg/kg/d of E2 and only 133 genes regulated by SPI plus E2 compared with their expression in the CAS group (Figure 2A). Of these genes, 266 genes, (about 50%) were regulated in a similar fashion by both the SPI diet and E2 treatment in these prepubertal rats compared to casein. However, this decreased to 24 genes when the SPI+E2 group was compared to CAS (Figure 2A).

More detailed microarray data analysis for these genes showed different patterns of gene regulation by SPI, E2 and SPI+E2 (Figure 2B). The top twenty-five up-regulated genes, and all down-regulated genes by either SPI or E2 are shown in Figure 2C and 2D respectively. Interestingly, the major SPI up-regulated genes were also up-regulated by E2, but less robustly. For example, Snap25 (Synaptosomal-associated protein 25), a known molecule expressed on osteoblastic cells [19] was up-regulated by both SPI and E2. The SPI down-regulated genes were also down-regulated by E2. However, many E2 down-regulated genes were up-regulated by SPI-feeding. Strikingly, the combination of E2 treatment and SPI feeding antagonized most of the gene changes produced by either treatment alone. The detailed data has been deposited in a MIAME compliant database (E.g. ArrayExpress, GEO), and it is available to check on the MGED Society website with accession number GSE30862 http://www.ncbi.nlm.nih.gov/geo/query/acc.cgi?acc=GSE30862.

**Figure 3:**
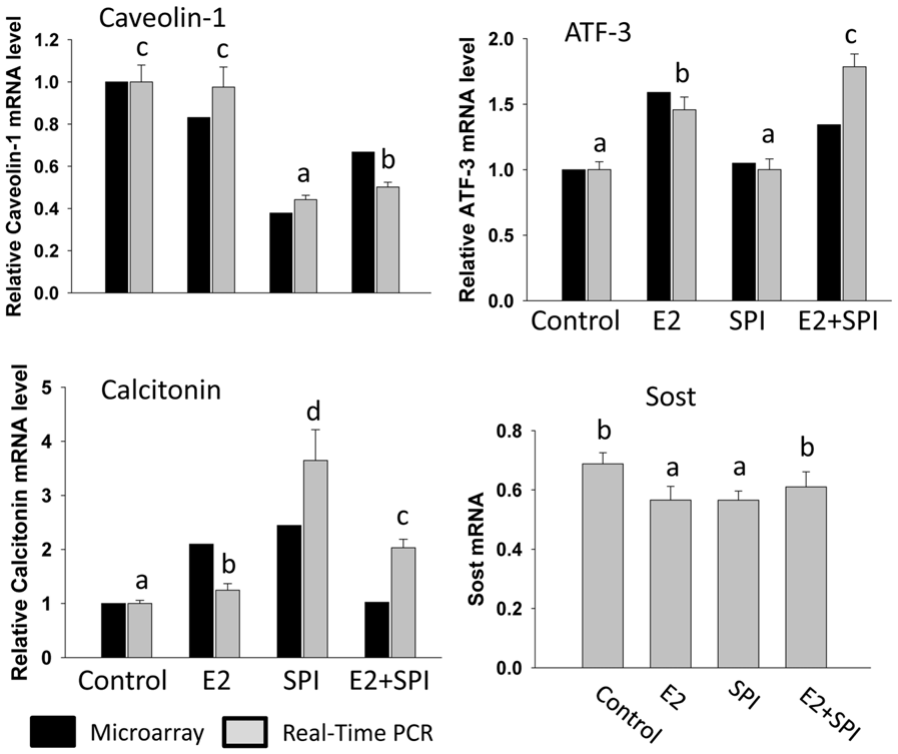
Real-time PCR verification of expression of genes of caveolin-1, ATF-3, calcitonin and Sost (sclerostin) in bone. Black bars represent fold changes with microarray analyses and gray bars represent relative mRNA expression from real-time PCR analyses. Control, standard casein diet group; E2, 10 µg/kg/d E2 treated group; SPI, soy protein isolate diet group; E2+SPI, combination of 10 µg/kg/d E2 treated and SPI diet group. Means ± S.E.M of real-time PCR results with different letters differ significantly from each other at p<0.05, a<b<c<d.

**Figure 4:**
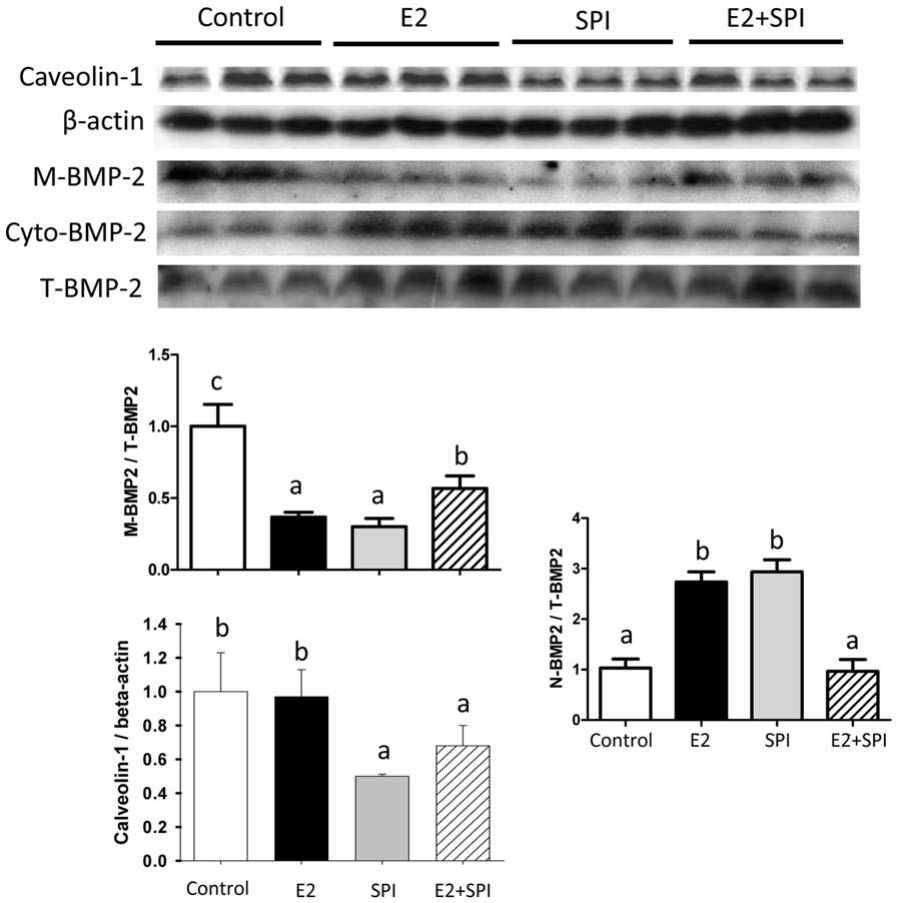
Caveolin-1, membrane versus cytoplasm BMP-2 protein expression in bone of pre-pubertal female rats from Control, standard casein diet group; E2, 10 µg/kg/d E2 treated group; SPI, soy protein isolate diet group; E2+SPI, combination of 10 µg/kg/d E2 treated and SPI diet group. Quantitation of the intensity of the caveolin-1 bands in the autoradiograms was performed relative to expression of β-actin, membrane BMP-2 (M-BMP-2) and nuclear BMP-2 (N-BMP-2) were performed relative to total BMP-2 (T-BMP-2). Data are Means ± S.E.M, n = 3, with different letters differ significantly from each other at p<0.05, a<b<c.

Analysis of the array data also demonstrated that the expression of mRNA encoding caveolin-1, a protein localized in the cell membrane, was down-regulated in rats fed SPI (2.8-fold), but not by E2 compared to CAS. This was confirmed by a real-time PCR analysis (p<0.05, Figure 3). On the other hand, expression of ATF-3 mRNA (activating transcription factor 3) known as a regulator of endochondral bone growth and skeletal development was only significantly up-regulated by E2 (both array and real-time PCR data ) and SPI did not block this effect (Figure 3). Calcitonin (calcitonin gene-related peptide) was up-regulated by both E2 and SPI (Figure 3) (both array and real-time PCR revealed) but to a much greater level by SPI than E2. Moreover, real-time PCR analysis showed decreases in mRNA expression of Sost (the gene encoding sclerostin) in bone after E2 or SPI treatment coincident with with decreases in the serum level of sclerostin presented in Figure 1 (Figure 3). Proteins were isolated from bone, and Western blots were performed. We found that down-regulation of caveolin-1 by SPI was associated with increased expression of BMP-2 (bone morphogenetic protein -2), particularly cytosolic BMP-2 expression (Figure 4). E2 treatment did not down-regulate caveolin-1, but increased cytosolic expression of BMP-2 compared to CAS (p<0.05), indicating E2 activates BMP-2 by mechanisms different than SPI.

### Both SPI and E2 increased BMP-2 and Runx2 but as a result of different molecular signaling pathways

**Figure 5:**
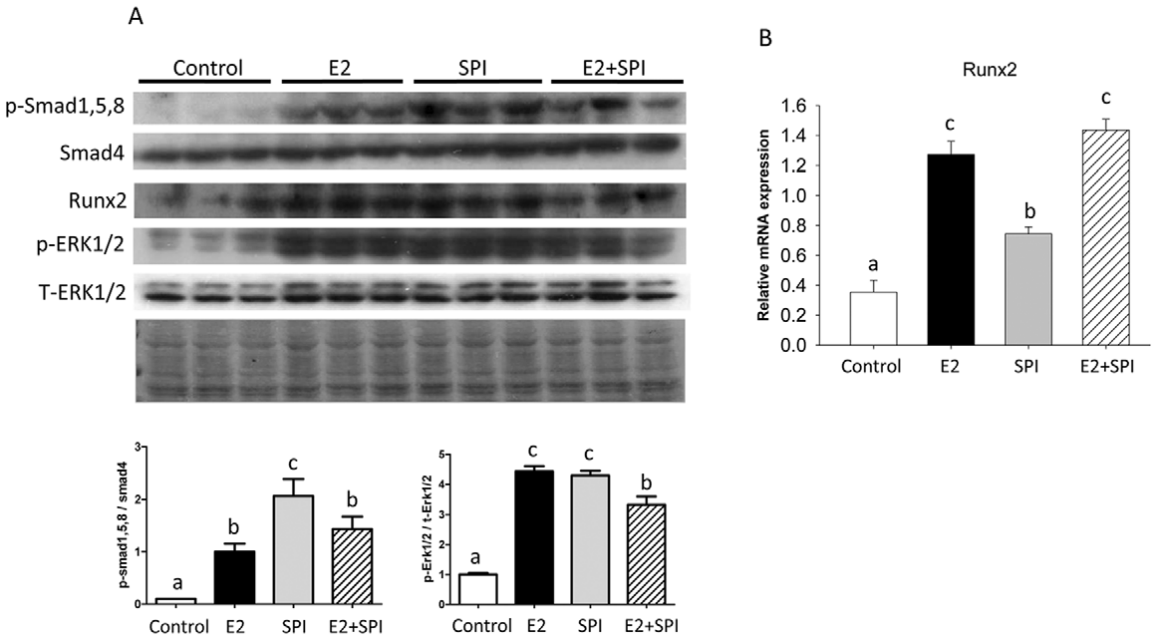
Cell signaling transduction pathway after activation of BMP-2 by SPI and E2 in bone. (A), Protein was isolated from bone after aspiration of bone marrow cells and performed Western blots. Smad and ERK1/2 phosphorylation and Runx2 protein expression in pre-pubertal female rat bone from Control, standard casein diet group; E2, 10 µg/kg/d E2 treated group; SPI, soy protein isolate diet group; E2+SPI, combination of 10 µg/kg/d E2 treated and SPI diet group. Quantitation of the intensity of the phosphorylated bands of p-Smad1, 5, 8 and p-ERK1/2 in the autoradiograms were performed relative to expression of total Smad4 and ERK1/2. Blot of Ponceau S staining showing protein loading control for western blotting. (B), Showing real-time PCR analyses for Runx2. Data are Means ± S.E.M, n = 3, with different letters differ significantly from each other at p<0.05, a<b<c.

**Figure 6:**
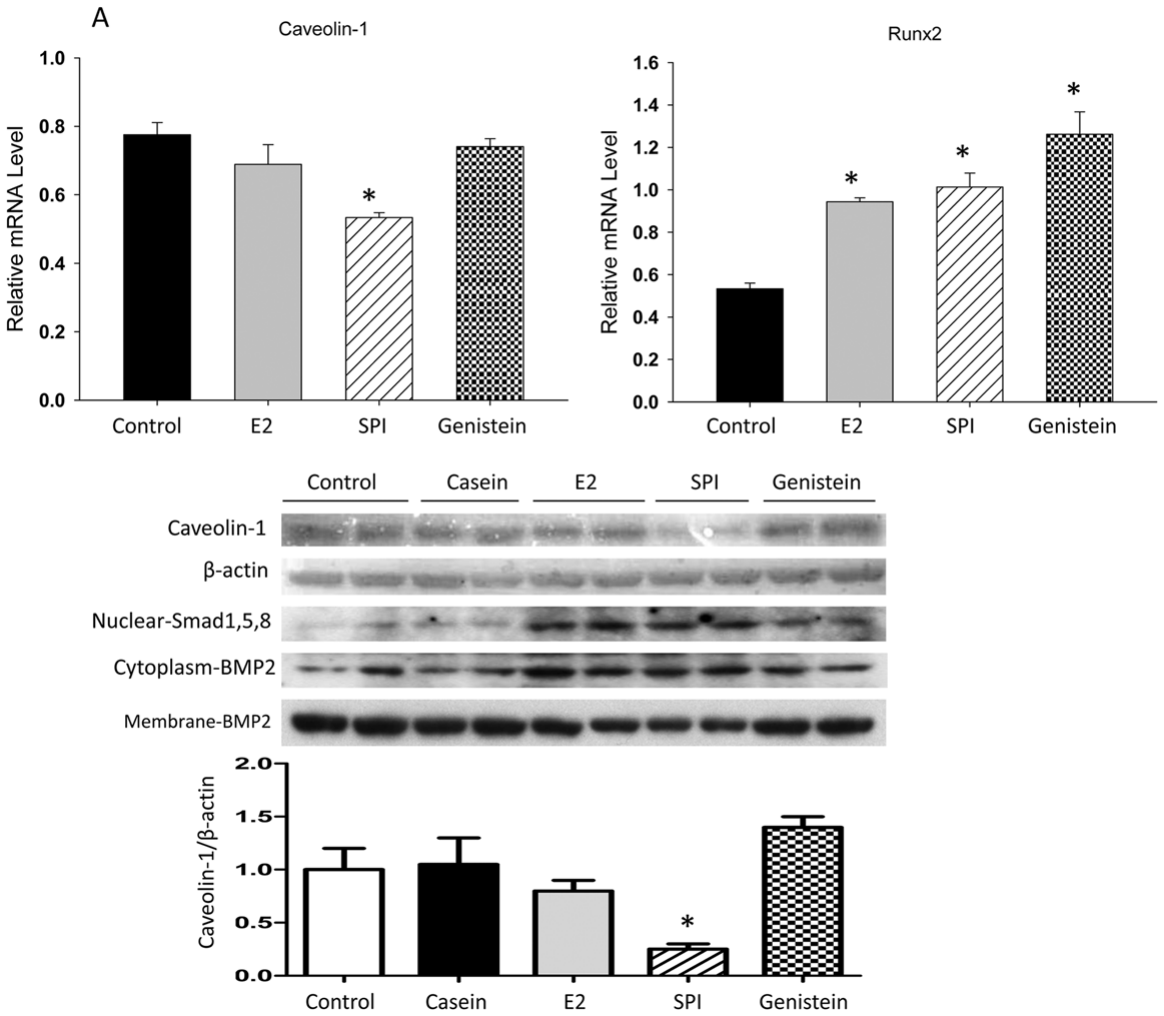
SPI stimulates osteoblast differentiation via down-regulation of caveolin-1. ST2 cells were treated with 2% serum either from control, 10 µg/kg/d E2 treated, SPI diet rats and 5 µM of purified genistein for 48 h. RNA and protein were collected. (A), Real-time PCR showing relative caveolin-1 and Runx2 mRNA expression. Data are Means ± SEM, triplicates, * p<0.05 versus control. (B), Western blotting showing caveolin-1 protein, nuclear SMD and cytosolic versus membrane BMP2 expression. Quantitation of the intensity of the caveolin-1 in the autoradiograms was performed relative to expression of β-actin.

It is known that the BMP-2 signaling cascade represents one of the most potent stimuli for bone formation. In order to determine cell signaling transduction after activation of BMP-2 by SPI, we isolated protein from bone after aspiration of bone marrow cells and performed Western blots. p-Smad 1/5/8, Runx2 and p-ERK were increased by both SPI and E2 treatment compared to the CAS group (p<0.05, Figure 5A). While real-time PCR analyses also showed significant up-regulation of Runx2 mRNA, the up-regulation by SPI was less than by E2 treatment or SPI plus E2 (p<0.05) (Figure 5B). Finally, we used an *in vitro* ST2 cell culture model to examine the differences between E2 and SPI on initiating osteoblastic cell differentiation signals. Cells were treated with 2% serum either from CAS control diet, 10 µg/kg/d E2 or SPI diet rats or with 5 µM of purified genistein for 48 h, RNA and protein were collected. Real-time PCR and Western blot analyses revealed that caveolin-1 was down-regulated (p<0.05) only by serum from SPI rats at both the mRNA and protein level (Figure 6). Down-regulated caveolin-1 was associated with increased cytosolic but decreased membrane expression of BMP-2 (Figure 6). Although serum from the 10 µg/kg/d E2 group and genistein both up-regulated Runx2 (mRNA) and BMP-2 (protein) expression, neither one regulated caveolin-1 at either the mRNA or protein levels (Figure 6). These findings demonstrate for the first time that SPI, genistein and E2 may all have some abilities to stimulate osteoblast differentiation, but the triggers of the signaling pathway are different.

## Discussion

Soy foods have been reported to have a variety of health benefits including prevention of cardiovascular disease and amelioration of bone loss in some clinical studies and animal models of osteoporosis [20]. At least one explanation for these beneficial effects is that soy foods contain weak estrogenic compounds – the isoflavones genistein and daidzein. However, the underlying mechanisms of soy diet or of purified isoflavones on bone and whether these effects are life stage-dependent remain unclear. Recent findings from a clinical osteoporotic patient trial showed that purified genistein has a very minor effect on bone added more controversy regarding the effects of soy diet on bone in the older population [21]. The results reported in our current study suggest that the effect of short term of SPI diet in pre-pubertal female rats on bone differed from that of classical estrogens. This SPI effect is consistent with our previously published data on rats and pigs [9], [17]. The effect of E2 on bone from our current study in pre-pubertal rats is also consistent with recent studies [22]. It is well known that estrogens have ability to suppress osteoclastic bone resorption leading to decreased bone remodeling and increased bone mass and strength. This bone remodeling suppressive effect of E2 was used for its clinical application on sex steroid deficiency-induced bone loss, because the hallmark of sex steroid deficiency-triggered bone loss is a high bone turnover. Compared with E2, bone turnover markers and microarray data indicated that the effect of soy diet on bone has some commonalities with E2. For example, they both decreased circulating levels of sclerostin and share regulation of some genes. However, the overall effect of the two treatments is decidedly different. Interestingly, the bone turnover data in SPI+E2 rats showed intermediary effects between SPI and E2 alone. The SPI and E2 combination group did not show additive effect of SPI on E2. This is consistent with our array data suggesting SPI and E2 may have mutually antagonistic effects on bone.

Relative to 17β-estradiol, serum isoflavone aglycone concentrations in SPI-fed rats are approximately 2200 times greater. However, because the potency of isoflavones is much lower than estradiol, estrogenic effects of isoflavones in soy diets on bone may be minimal or even antagonistic in the face of endogenous levels of estrogens. Therefore, the bone effects of soy diets may depend on developmental stage and endogenous E2 levels.

Consistent with our *in vivo* data, when we treated uncommitted osteoblast progenitors with serum from E2- or SPI-treated animals, we found that serum from SPI rats significantly down-regulated caveolin-1 at both the mRNA and protein levels. In sharp contrast, both E2 and genistein did not down-regulate caveolin-1. These data combined with the gene expression data suggest that a dietary factor(s) other than isoflavones appearing in serum after consumption of soy diets acts on caveolin-1 expression via an estrogen-independent pathway. It has been reported that increased trabecular bone is one of the phenotypes in caveolin-1 deletion mice [23]. Caveolin-1 is a scaffolding protein containing a 33 amino acid hydrophobic domain that anchors the protein to the membrane [24]. Such proteins include bone morphogenetic proteins (BMP). Our *in vivo* data support the hypothesis that down-regulation of caveolin-1 triggers release of BMP-2 from the membrane, activating associated kinases in the cytoplasm. These data suggest that the effect of soy diets on osteoblastogenesis is largely due to actions via BMP signaling. On the other hand, BMPs also activate intracellular transcription factors Smad-1, -5 and -8 (Smad-1/5/8) and proceed toward dimerization with Smad-4 (Co-Smad, common Smad) before translocation into the nucleus [25]. Nuclear translocated Smad complexes then regulate bone specific gene transcription. BMPs have also been reported to stimulate MAP kinase, supporting the idea that Smad-independent signals may be required for the maximum activation of BMP signals in osteoblasts [26]. All of those above mentioned BMP-associated transduction molecules, as well as Runx2, an absolute osteoblast differentiation transcription factor, were activated in bone tissue from SPI-fed animal *in vivo*. These results suggest novel actions of soy diet on bone development. E2 shares part of the described signaling, however, it did not initiate activation of caveolin-1 and thus may act through activation of its own receptors, estrogen receptor alpha or beta.

In summary, we have utilized pre-pubertal female rats as a model and demonstrated distinct effects of E2 and SPI diet on bone even though both treatments seem to share some pathways. E2 plus SPI had no additive effects on bone but rather the two treatments cancelled each other out suggesting negative cross talk between SPI-regulated and E2-regulated pathways. Consumption of the SPI diet down-regulated the expression of caveolin-1, and was associated with increased cytosolic BMP2, and Smad and Runx2 expression in bone and osteoblasts. These results suggest that SPI consumption results in significant stimulation of osteoblastogenesis prior to puberty, and initiation of osteogenic signals may differ than estrogen at this developmental stage.

## Materials and Methods

### Animal Experiments

Time-impregnated female Sprague-Dawley rats (Harlan Industries, Indianapolis, IN) arrived on gestational day 4 and were individually housed in an Association for Assessment and Accreditation of Laboratory Animal Care-approved animal facility at the Arkansas Children's Hospital Research Institute with constant humidity and lights on from 06:00–18:00 hrs at 22°C. All animal procedures were approved by the Institutional Animal Care and Use Committee at University of Arkansas for Medical Sciences (UAMS). The approval ID for this study is 2473. Pregnant rats were fed AIN-93G diets made with casein as the sole protein source [27] and their female offspring weaned at postnatal day 20 (PND20) onto one of the following diets: AIN-93G diets with casein (CAS) or made with soy protein isolate (SPI). The diets were made to be isonitrogenous and isocaloric. The calcium and phosphorus levels were adjusted to be the same in each diet. Diets were made according to the AIN-93G diet formula, and the protein source was either casein (Fonterra, Santa Rosa, CA) or SPI (Clinical blend 670, a gift from Solae, Du Pont, St. Louis, MO). The percentage of protein content of each diet was 20%. Amino acids were added to each diet to equalize the essential amino acids among diets. The detailed composition of the SPI diet was fully described in our lab previously [28]. Two groups of rats received E2 administered by Alzet mini osmotic pump (model # 2004, AlzaCorp., CA) implanted subcutaneously. The dose was 10 µg/kg/day of E2. After 14 d of *ad libitum* feeding, the rats were sacrificed 1 hour following lights-on by injection with 100 mg Nembutol/kg body weight (Avent Laboratories), followed by decapitation. Blood and bone (tibia) were collected.

### Serum Bone Turnover Markers and Isoflavones

The serum bone formation marker alkaline phosphatase (ALP) was measured by a colorimetric assay using the time-dependent formation of p-nitrophenolate from para-nitrophenyl phosphate (PNPP). The protocol used 5 µl of serum in a total volume of 1.5 ml containing 0.05 mmol/L PNPP, 2 mmol/L MgCl2, and 10 mmol/L L-phenylalanine (to inhibit any circulating intestinal ALP activity). The serum bone formation marker osteocalcin and the serum bone resorption marker RatLaps (bone-related degradation products from C-terminal telopeptides of type 1 collagen) were measured by Rat-MID™ Osteocalcin ELISA and RatLaps™ ELISA, respectively, from Nordic Bioscience Diagnostics A/S (Herlev Hovedgade 207, 2730 Herlev, Denmark). Aglycones and conjugated serum isoflavones were extracted and analyzed as previously described [18]. Briefly, aglycones were measured directly by LC-MS, and conjugated isoflavones were enzymatically deconjugated by incubating serum (100 µl) with a mixture of sulfatase and glucuronidase (100 U Sulfatase H-5, Sigma) and were measeured by LC-MS to obtain total isoflavones. Samples were also incubated with b-glucuronidase (1000 U, B-1, Sigma) to obtain the sum of glucuronidases and the aglycones. Isoflavone sulfates were calculated by subtracting the glucuronidase and aglycones from the total concentration.

### Gene Microarray and Real-Time Reverse Transcription-Polymerase Chain Reaction (Real-time RT-PCR)

After aspiration of bone marrow, right tibial bone total RNA was extracted using TRI Reagent (MRC Inc., Cincinnati, OH) according to the manufacturer's recommendations followed by DNase digestion and column cleanup using QIAGEN mini columns. Three microarrays (GeneChip Rat 230 2.0; Affymetrix, Santa Clara, CA) were used for each diet group; control, SPI, E2 and SPI plus E2 treated animals. Pools of equal amounts of RNA from two to three rats were used for analyses per microarray, representing at least eight rats per group over the three microarrays. cRNA synthesis, labeling, hybridization, and scanning were carried out using the manufacturer's instructions and described previously in our lab [29]. Microarray data analyses were carried out using GeneSpring version 7.3× software (Agilent Technologies, Santa Clara, CA) [29]. The CEL files containing probe level intensities were processed using the robust multiarray analysis algorithm for background adjustment, normalization, and log2 transformation of perfect match values [29]. Subsequently, the data were subjected to normalization by setting measurements less than 0.01 and by per-chip and per-gene normalization using GeneSpring. The normalized data were used to generate a list of differentially expressed genes among different diets with and without E2. The detailed data has been deposited in a MIAME compliant database (E.g. ArrayExpress, GEO), and it is available to check on the MGED Society website with accession number GSE30862 http://www.ncbi.nlm.nih.gov/geo/query/acc.cgi?acc=GSE30862. Real-time RT-PCR was carried out using SYBR Green and an ABI 7000 sequence detection system (Applied Biosystems, Foster City, CA). Primers for rat and mouse caveolin-1, ATF3, calcitonin and Runx2 were designed using Primer Express software 2.0.0 (Applied Biosystems). Their sequences are all listed in Table 2.

**Table 2:** Real-Time Reverse-Transcription Polymerase Chain Reaction (RT-PCR) Primer Sequences.

Gene	Forward Primer	Reverse Primer
Rat		
Caveolin-1	GAGTCTGCCAAAGCAAGATTGCCA	AGGCTTCGCAGCGTTACAGACTAT
RUNX2	CCGTGGCCTTCAAGGTTGTA	ATTTCGTAGCTCGGCAGAGTAGTT
ATF-3	TTTCAAAGGGCGTAGGACTCCACA	TTCAAATACCAGTCTCCACGGGCT
Calcitonin	AGAAGAGATCCTGCAACACTGCCA	GGCACAAAGTTGTCCTTCACCACA
Sost (Sclerostin)	GGCAAGCCTTCAAGAATGATGCCA	TGTACTCGGACACGTCTTTGGTGT
GAPDH	TGAGGTGACCGCATCTTCTTG	TGGTAACCAGGCGTCCGATA
Mouse		
Caveolin-1	TGTACCGTGCATCAAGAGCTTCCT	GTGCTGATGCGGATGTTGCTGAAT
RUNX2	CGGTCTCCTTCCAGGATGGT	GCTTCCGTCAGCGTCAACA
GAPDH	GTATGACTCCACTCACGGCAAA	GGTCTCGCTCCTGGAAGATG

### *In vitro* Osteoblast Differentiation Cell Culture

For *in vitro* cell culture experiments, bone marrow stromal cell line ST2 cells were utilized and treated with serum from different treatment groups. Cells were cultured in the plates in the presence of minimal essential medium (Invitrogen, Calsbad, CA) with 10% fetal bovine serum (FBS) (Hyclone Laboratories, Logan, UT) and 100 U/ml each of penicillin and streptomycin (Sigma-Aldrich). When actual experiments were started, cells were cultured with 8% FBS without osteogenic medium as negative control. The effects of treatment with 5% FBS plus either 2.0% 0.45 µm filtered serum from control, SPI- or E2-treated rats or purified genistein on ST2 cells were compared. Cell RNA and protein isolations and real-time RT-PCR, western blotting were performed similarly with the method described above.

### Western Blotting

Right tibial bone tissue proteins for Western immunoblot analysis were extracted using cell lysis buffer as described previously [9]. Membrane and nuclear protein isolations were performed according to procedures provided by manufacturer (PIERCE Biotechnology). The phosphorylation status of ERK1/2 was examined by Western blotting using a mouse monoclonal antibody recognizing tyrosine phosphorylated ERK1/2 or rabbit polyclonal antibodies recognizing total ERK1/2 (Santa Cruz Biotechnology) followed by incubation with either an anti-mouse or an anti-rabbit antibody conjugated with horseradish peroxidase (Santa Cruz Biotechnology). Similar Western blotting studies were performed with antibodies to caveolin-1 (Santa Cruz Biotechnology), BMP2 (Cell Signaling), Smad 4 and phospho-Smad 1/5/8 (Cell Signaling), and Runx 2 (Santa Cruz Biotechnology). Primary antibodies were detected with a horseradish peroxidase-conjugated secondary antibody (Santa Cruz Biotechnology). Blots were developed using chemiluminescence according to the manufacture's recommendations. Quantization of the intensity of the bands in the autoradiograms was performed using a VersaDoc™ imaging system (Bio-Rad).

### Data and Statistical Analyses

Data were expressed as mean ± SEM One way analysis of variance (ANOVA) was utilized to analyze diet effects and two-way ANOVA for analysis of SPI, E2 interactions followed by student Newman-Keuls post hoc analysis for multiple pair-wise comparisons between treatment groups. Values were considered statistically significant at p<0.05.
